# Finding the Sweet Spot: Preferences for Effectiveness, Duration, and Side Effects in a Discrete Choice Experiment Among Uganda’s Key Populations

**DOI:** 10.3390/vaccines13111090

**Published:** 2025-10-24

**Authors:** Maiya G. Block Ngaybe, Richard Muhumuza, Mélanie Antunes, Ezra Musingye, Kawoya Kijali Joseph, Betty Nakaggwa, Stephen Mugamba, Bashir Ssuna, Gabriela Valdez, John Ehiri, Maia Ingram, Agnes Kiragga, Grace Mirembe, Betty Mwesigwa, Hannah Kibuuka, Purnima Madhivanan

**Affiliations:** 1 Department of Health Promotion Sciences, Mel and Enid Zuckerman College of Public Health, University of Arizona, 1295 N Martin Ave., Tucson, AZ 85721, USA; gvaldez@arizona.edu (G.V.); jehiri@arizona.edu (J.E.); maiai@arizona.edu (M.I.); pmadhivanan@arizona.edu (P.M.); 2 Medical Research Council, Uganda Virus Research Institute and London School of Hygiene and Tropical Medicine Uganda Research Unit, 51/59 Nakiwogo Rd, Entebbe P.O. Box 49, Uganda; richmuhm@gmail.com; 3 The Health Economics Research Unit, University of Aberdeen, King’s College, Aberdeen AB24 3FX, UK; m.antunes.19@abdn.ac.uk; 4 Makerere University Walter Reed Program, Plot 42 Nakasero Rd, Kampala P.O. Box 16524, Uganda; emusingye@muwrp.org (E.M.); kawoyakijjalijoseph@gmail.com (K.K.J.); nakaggwabetty@gmail.com (B.N.); smugamba@muwrp.org (S.M.); gmirembe@muwrp.org (G.M.); bmwesigwa@muwrp.org (B.M.); hkibuuka@muwrp.org (H.K.); 5 Uganda Tuberculosis Implementation Research Consortium (U-TIRC), Kampala P.O. Box 21696, Uganda; bashir.ssuna@aya.yale.edu; 6 Infectious Disease Institute, Kampala P.O. Box 22418, Uganda; akiragga@aphrc.org; 7 Public Health Research Institute of India, Mysore 570020, Karnataka, India

**Keywords:** HIV prevention and control, vaccination, HIV vaccine, broadly neutralizing antibodies, PrEP, health economics, discrete choice experiment

## Abstract

**Background:** Human immunodeficiency virus (HIV) affects more than 39 million people worldwide, with Uganda ranked 10th among countries with the highest number of cases. As new preventative HIV injectables emerge, it is vital to think about how best to tailor strategies to promote these injectable drugs, like PrEP and vaccines, when available, to the different populations most in need. Discrete choice experiments (DCEs) are economics-derived methods used to determine factors that influence engagement in a certain behavior. **Objective:** This study used a DCE to determine the preferences for a preventative HIV injectable drugs/vaccines among people at risk of HIV acquisition in urban and peri-urban areas of Uganda. **Methods:** In June 2024, we implemented a cross-sectional DCE survey in three urban sites in Uganda in English and Luganda. The survey collected information on demographics, HIV risk, vaccine confidence and responses to the 13 injection product choice tasks presented to determine preferences. We used community-based, respondent-driven sampling methods to recruit participants from three key populations: (1) female sex workers; (2) people who identify as lesbian, gay, bisexual or transgender; and (3) young women (18–24 years). We collected the data on tablets using the Sawtooth Lighthouse Studio software (v. 19.15.6), taking into consideration privacy and confidentiality, given the sensitivity of the information and recent governmental policies in Uganda. Data were analyzed using a split-sample mixed logit regression analysis. The study was approved by local ethical regulatory bodies. **Results:** From the total of 406 participants screened for this study, 376 participants met the eligibility criteria and were included in the final analysis (85 young women, 159 female sex workers, and 132 who identified as lesbian, gay, bisexual or transgender). The average age was 23.7 (SD: 5.7). The majority of participants had received some secondary school or vocational school (202, 53.7%) The attributes that explained the preferences were primarily severe compared to mild side effects (*β*: −0.69, 95% CI: −0.78, −0.60), a 30% increase in vaccine/drug effectiveness (*β*: 0.39, 95% CI: 0.34, 0.44), and a 50,000 UGX (or USD ~13.64) increase in cost (*β*: −0.22, 95% CI: −0.27, −0.17). There were no significant differences between the preferences for different injectable types. The sensitivity analyses suggested potential differences in preferences by the amount of help participants received from research assistants when completing the survey, although not by income level. **Conclusions:** Side effects had the greatest impact on participants’ preferences for injectable HIV prevention methods, followed closely by effectiveness and cost. It is therefore essential to develop affordable or free prevention options with minimal side effects. Policymakers should focus on reducing the financial barriers to access and emphasize transparent communication about the effectiveness and safety of these injectables in health promotion campaigns to maximize adoption and improve public health outcomes.

## 1. Introduction

Human immunodeficiency virus (HIV) affects more than 39 million people worldwide [1]. Uganda is ranked as the 10th country with the highest number of people living with HIV [2]. Uganda has a prevalence of 5.1% and an incidence of 0.22% as of 2022/23 [1]. Key populations, such as sex workers, young women and lesbian, gay, bisexual and transgender (LGBT) groups, are especially at risk of contracting HIV and need reliable protection from the infection [1]. A study in 2018 found the highest rates of HIV and other sexually transmitted diseases to be among the age group of 18–30-year-olds, indicating that young adults may be most at risk [3].

Of the 23,440,016 people who identified as female in Uganda in 2024, 2,284,081 (~10%) were between 19 and 24, representing young women [4]. According to the most recent estimates of key population sizes in Uganda reported in 2019, women engaged in sex work between the ages of 15 and 49 years were estimated to represent about 1.49% of the population (130,359 total) [5]. Men who have sex with men above the age of 15 were estimated to comprise around 0.25% of the population (22,663 total) [5].

There is a growing variety of new products being developed for HIV prevention to encourage a larger proportion of people at risk of contracting HIV to protect themselves. Long-acting products, especially injections, appear to show a lot of promise to be widely desirable among these populations, as they would reduce the burden of frequent doses and associated inconveniences like transportation costs or stigma experienced by methods like oral pre-exposure prophylaxis (PrEP) [6]. Proposed long-term preventative options include implants, injections, and vaccines [7]. Some social challenges make it especially difficult to arrange access to care for these populations. However, longer-term preventative options like an injection may make it easier for key populations to reduce their risk of HIV acquisition and to overcome social challenges, such as stigma and the dangers that may accompany seeking preventative services [8]. With the recent release of long-acting PrEP injections, new innovative HIV vaccine Discovery Medicine clinical trials, and the promise of the preventative ability of broadly neutralizing antibodies, many key populations are demonstrating readiness for long acting injections to protect themselves [9,10]. Recent discrete choice experiments (DCEs) conducted in South Africa and Kenya among young women and key populations confirm the high desirability of injectable PrEP formulations, particularly when injections offer less frequent dosing, privacy, and lower perceived stigma compared to daily oral regimens. These findings underscore the importance of user-centered design and attribute-based preference elicitation when developing or scaling up long-acting HIV prevention options in African settings similar to Uganda [11,12].

The acceptability of long-acting PrEP has been widely demonstrated around the world, particularly in Uganda, with uptake rates of 70% among high-risk young women [13] and 64% among key populations [9]. While there is little research in Uganda on willingness to vaccinate against HIV, there have been several studies that have assessed willingness to participate in HIV vaccine trials, as Uganda is one of the first locations in Africa to engage in HIV vaccine trials [14]. Previous research in Uganda focusing on willingness to participate in HIV vaccine trials has mostly found a high level of willingness (77–95%) to participate in the HIV trials [15,16,17,18,19,20]. However, this willingness was found to be curbed by concerns about vaccine safety, blood draws, and time required to participate, and increased by concerns of infidelity of current sex partners [15,21]. There is no research to date on the acceptability of injectable broadly neutralizing antibodies. While needle aversion is common, interest and willingness to utilize injectable options remain high [22,23]. However, little is known about how people may compare different injectable options or about their preferences for an injectable long-acting preventable HIV product.

One effective methodology for exploring preferences, especially for products that have not yet been released, is the Discrete Choice Experiment (DCE), a form of stated preference study. The DCE methodology is based on the economic theory of preference elicitation, specifically stated preference methods [24]. According to consumer theory in economics, individuals are assumed to be rational decision makers in search of a maximized and stable set of preferences [24]. According to Lancaster’s economic theory of value, individuals are assumed to derive utility not simply from the good or service itself, but instead from the underlying attributes of the end service or good of interest, and preferences are thereby revealed through the evaluation of these attributes [25]. In a DCE, participants are asked to trade off the costs and benefits of each characteristic of different alternatives, which are presented within a scenario, and in doing so, they demonstrate their preferences. This approach has been successfully applied in recent studies in sub-Saharan Africa to understand preferences for injectable PrEP among young women and other key populations, offering valuable precedent for using DCEs in HIV prevention research. These studies revealed that dosing interval, clinic visit frequency, and confidentiality of use were among the most influential attributes shaping decisions around injectable product uptake [11,12].

This study aimed to answer the question of which factors are most preferable among populations at increased risk for HIV and therefore influence their decisions to take up a preventative HIV injection, through the implementation of a DCE in Uganda. This study addresses an important gap in the literature, namely the comparison of the valuation of the injectable PrEP product compared to an HIV vaccine. Should participants value an HIV vaccine, it may demonstrate its added benefit to the participants and justification of continued research of said product.

## 2. Materials and Methods

### 2.1. Study Setting

The Makerere University Walter Reed Program (MUWRP) is a biomedical research organization located in Kampala, Uganda, with a long history of vaccine and therapeutic research in infectious diseases. The study was conducted in June 2024 at three MUWRP sites in urban and peri-urban Uganda, specifically in Kampala, Mukono and Kayunga.

### 2.2. Study Population

The study population of interest included (1) women in sex work; (2) people who identify as lesbian, gay, bisexual or transgender; and (3) young women (aged 18–24 years). Participants were only included if they were over the age of 18 years, were able and willing to provide informed consent, were available to participate in the survey, and understood English or Luganda. These participants were purposively sampled with the help of the MUWRP Community Engagement team to gather data from diverse sources using the MUWRP existing networks from previous HIV studies. Participants were purposefully sampled from subgroups of interest identified through known community “hot spots.” Recruitment was facilitated through peer referral within each location to enhance trust and access. To ensure geographic representation, participants were enrolled from multiple sites across Kampala. Demographic distributions were continuously monitored throughout recruitment, allowing for iterative adjustments to maintain sample diversity and balance. Sampling strategies were strengthened by consulting with experienced community engagement team members, the community advisory board and peer leaders in the community to ensure that participants felt comfortable and safe, and that conversations were private.

### 2.3. Sample Size

The sample size was determined a priori based on Orme’s rule of thumb: at least 111 for each of the three subgroups recruited for the study, aiming for 125 per group considering the risk of loss of data for any reason [26].

### 2.4. Discrete Choice Experiment

The DCE survey for this study was designed based on a literature review, expert consultations and key informant interviews among HIV prevention experts and key populations in Uganda. The key informant interviews are described in more detail in another manuscript. Attributes and levels were determined based on characteristics of possible future injectable HIV prevention options that were either recently released or in development, those that mattered most to members of the target population in the qualitative interviews, as well as characteristics that could feasibly be implemented.

Three different preventative injectable products, either in development or recently released, were chosen to be included in the DCE based on expert consultations: a preventative HIV vaccine, long-acting injectable PrEP, and broadly neutralizing antibody injections. A local artist in Kampala collaborated with the research team to design relevant graphics for each level, to facilitate participants’ engagement with the choice tasks. Other attributes were included based on a systematic literature review of relevant stated preference studies, qualitative research and further expert consultation. Levels were further refined based on the pilot study (see Table 1).

### 2.5. Experimental Design

The DCE survey measures preferences across a range of combinations of levels for each attribute by presenting respondents with different feasible HIV preventative injectable options and observing their stated choices between those options. In a DCE, the experimental design determines the specific block, or group of choice tasks, presented to each respondent. Lighthouse Studio Software (v 9.15.6) was used to select a balanced overlap experimental design that minimized the standard errors, generating a total of 300 blocks, with 13 choice tasks in each block and 2 alternatives per choice task. No constraints were imposed in the selection of choice tasks for the experimental design.

### 2.6. Choice Task

**Figure 1:**
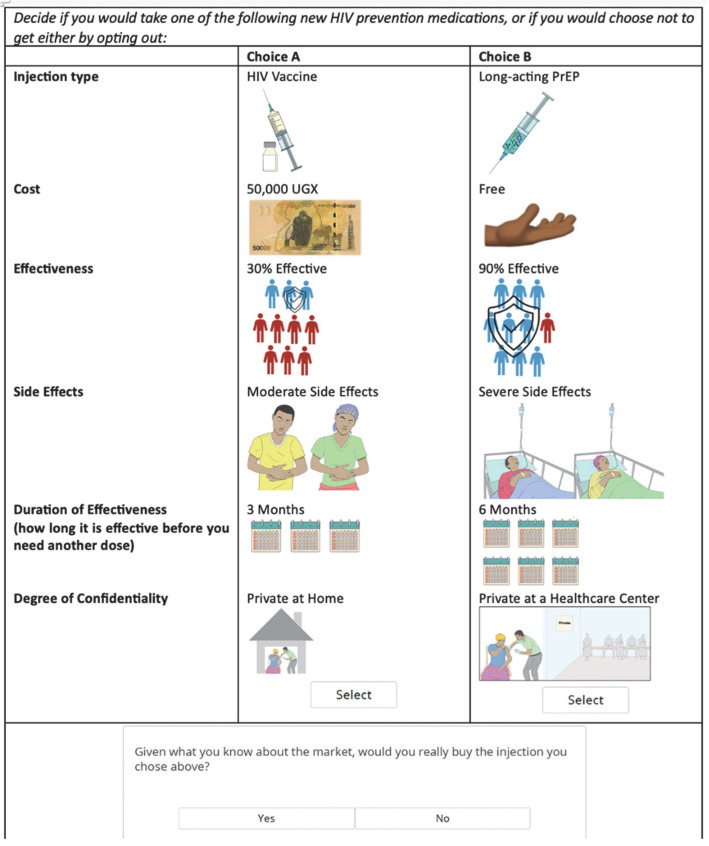
Choice task example from the HIV preventative injectable discrete choice experiment.

In the DCE choice task, each participant was asked to decide if they would take one of the “following new HIV prevention medications,” or if they would choose not to get either by opting out. This was structured as a two-step question. Participants were first asked to choose between two options, choice A and choice B, before continuing to a secondary opt out question (or None question). In this second question, participants were asked if they would really purchase the option they had selected if faced with the decision in real life (Figure 1).

### 2.7. Covariates

Participants were asked to answer demographic questions: marital status, ability to read and write, highest level of education completed, employment status, religion, tribe, age, sex/gender, number of people in the household, number of people they provide for financially and household income. They were also asked questions related to their HIV risk: including if they know someone living with HIV and their relationship with that person, their level of concern about contracting HIV and whether they inject drugs. In addition, participants were asked if in the last six months they: have been sexually active, have had anal sex, used condoms consistently during sex, have had sex with more than one partner, have had an STI, have taken PEP post-exposure to HIV, or have shared injecting materials. An open-ended question asked participants which HIV prevention methods they had used in the last week.

The participants were also asked to answer the Vaccine Confidence Index [27,28] (VCI) questionnaire, which consisted of four statements rated on a 5-point Likert scale ranging from “Strongly Agree” to “Strongly Disagree”. Finally, the Research Assistant was asked to report any additional notes relevant to the survey and report the degree of help they provided the participant (“I did not help the participant complete the survey,” “I helped the participant a little,” “I helped the participant a lot”). A full list of all questions and their response options is included in the questionnaire attached as Supplementary Table S1.

For the analysis, some categories were condensed to avoid sparse categories, and the subgroup membership was an important variable for the analysis. Although participants did at times belong to multiple groups, for the analysis, any young woman aged 18–24 years was excluded from the young woman group if categorized as part of the female sex worker subgroup. Similarly, participants who identified as LGBT and as either sex workers or young women were categorized within the LGBT subgroup.

### 2.8. Data Collection

Participants were invited to a mutually agreed upon confidential location. After providing informed consent, participants were asked to complete a demographic questionnaire (see Appendix A Table A2). Participants were deemed eligible if they were above the age of 18 and fell under one of the three subpopulations of interest: young women (aged 18–24), identifying as lesbian, gay, bisexual or transgender (LGBT), or women in sex work (defined as exchanging money for sex and identifying as female). The demographic and eligibility questionnaires were administered on a tablet or paper if the tablet was not available. The interviewers then proceeded with data collection.

Participants were first shown a preamble crafted to explain and define the task, attributes and levels. There was a special emphasis on explaining the difference between different injectable types, since participants in the preceding qualitative phase had emphasized the need for more information about the injections.

All study materials were translated into Luganda and back translations were obtained for key parts of the text identified as problematic during the pilot study. The pilot study was conducted with 12 participants with read-aloud interviews to validate the survey, ensure translations were accurate and understandable by the participants, assess respondent burden, and test the timing of the survey.

All participants provided written consent before engagement in any study activities. The study was approved by the Makerere University School of Public Health Research and Ethics Committee and the Uganda National Council for Science and Technology (SPH-2023-465/HS 3769ES).

### 2.9. Data Analysis

A descriptive analysis was conducted for subgroups to summarize demographics and HIV prevention practices. For this analysis, participant responses were reported as raw counts with frequency percentages, or as means with standard deviations, by subgroup and for the full sample. For the VCI, we reported the means and standard deviation for each subgroup and the total sample. We reported the mean Index score as the mean of all items’ scores for each subgroup. We ran tests of normal distribution for all continuous variables when comparing subgroups. We used an ANOVA to compare means, and the Pearson Chi-square test or Fisher’s exact test when cell frequencies were less than 5 to compare proportions between groups, with statistical significance defined as a *p*-value of less than 0.05.

We analyzed the choice problem using a mixed logit regression to understand how attributes included in the discrete choice experiment are associated with the choice for a preventative HIV injection. In this analysis, each choice was not assumed to be independent in order to account for the panel structure of the data and relax the assumption of independent and identically distributed errors in the model. This approach also allowed us to explore unobserved heterogeneity. We only considered the Alternative Specific Constant (ASC) parameter as random and included all other parameters as fixed. When testing the model, our limited sample size could not support the estimation of additional random parameters beyond the ASC. We selected the error-components mixed logit to capture unobserved heterogeneity in uptake via a random ASC, while keeping attribute coefficients fixed for interpretability and cross-group comparison. Including additional random parameters did not improve model fit (as assessed via log-likelihood, AIC/BIC) due to non-significant standard deviations and model instability. The number of iterations for the mixed logit regression was limited to 500, and the ASC distribution was assumed to be normal.

A split-sample analysis was conducted to predict preferences across the three at-risk subgroups that were pre-determined based on the qualitative phase, the literature review and expert consultations: young women, sex workers and sexual minority groups (lesbian, gay, bisexual, and transgender). We ran a likelihood ratio test to determine if there were significant differences between the three groups before subgroup analysis.

We then calculated marginal willingness to pay in USD for the alternatives and levels included in the DCE using the Delta Method. Prospective uptake rates were also estimated for four different preventative injection options using the DCE mixed logit regression results. The four injections included two recently released long-acting PrEP injection products: cabotegravir (CAB-LA) and lenacapavir, and hypothetical HIV vaccine and broadly neutralizing antibody (bNAbs) injection products.

### 2.10. Sensitivity Analysis

Some participants were allowed to complete the task by themselves, while others required assistance, which may have compromised the survey quality for participants who received less help (e.g., reading aloud questions or helping to explain words). Although all questions were forced choices and research assistants monitored to ensure participants reached the final page to avoid missing data, responses were omitted from the final analysis if participants left early for any reason and data were incomplete. To explore potential bias related to the amount of help received, we conducted a sensitivity analysis using a likelihood ratio goodness of fit test to compare the conditional logit regression across subgroups based on the level of assistance. We also tested if economic status impacted the study’s results through likelihood ratio goodness of fit test, comparing two subgroups based on a bivariate income level (participants who make less than or equal to 50,000 UGX compared to those who make more than that amount). All data analyses were conducted using the programs Stata (v18) and Lighthouse Studio (v 9.15.6).

The DIRECT checklist for reporting on DCEs was used to guide the reporting of our findings in this manuscript [29].

## 3. Results

### 3.1. Demographic Characteristics

We screened 406 participants for this study, but only 376 (92.6%) participants were eligible and enrolled. Participants included 85 young women aged 18–24, 159 female sex workers, and 132 who identified as lesbian, gay, bisexual or transgender (LGBT). Most participants recruited were from Kampala (83.2%). The highest proportion of participants preferring to take their survey in Luganda was among Young Women (60.0%).

The average age of participants was 23.7 (SD: 5.7). The majority of participants identified as female (72.9%, 274), but most LGBT participants identified as male (58.3%), followed by female (22.7%). Marital status was primarily single across all groups (77.7%), followed by married (14.4%).

Most participants reported being able to read and write (95.2%), though the highest proportion was among LGBT participants (98.5%). Most participants had received at least some secondary or vocational school education (53.7%). LGBT participants tended to report higher education levels with 14.4% having completed some university degree, compared to 6.3% and 5.9% among female sex workers and young women respectively. About three-quarters (75.5%) of all participants reported being employed. The reported weekly income in Ugandan shillings (UGX) was on average 85,828 UGX (SD: 106.1 K) (Table 2). More than half of participants (233/376, 62%) agreed that they would get the injection in real life.

### 3.2. HIV Risk Characteristics

**Figure 2:**
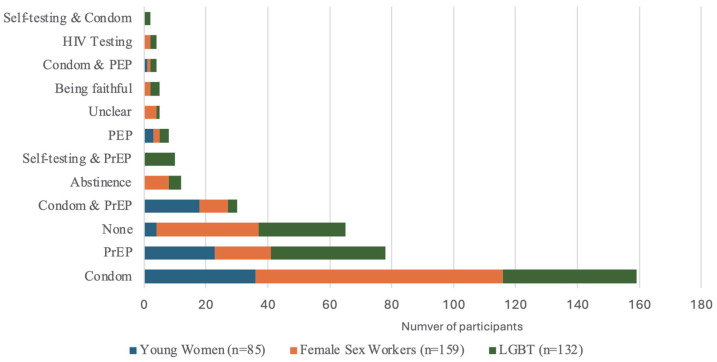
HIV prevention method used in last week among DCE participants by subgroup, *n* = 378.

Participants primarily reported using only condoms as their HIV prevention method in the last week (42.3%), although usage was highest among female sex workers (50.3%), followed by young women (42.4%) and then LGBT groups (32.6%). The next most reported HIV prevention method was PrEP by itself (20.7%), although its highest usage was reported among LGBT (28.0%) and young women (27.1%), compared to female sex workers (11.3%) (Figure 2).

An average of 76.6% of participants knew someone living with HIV. Most participants were concerned about getting HIV (88.3%). Most participants reported being sexually active in the last six months (93.6%). Three-quarters of LGBT participants (75.0%) reported having anal sex in the last six months, while female sex workers (5.7%) and young women (5.9%) mostly did not. Less than half of participants (41.8%) reported regularly using condoms in the last six months. About two-thirds (68.9%) of participants reported having more than one sexual partner in the last six months, with the highest rates among young women (94.1%). About half of participants reported having had a sexually transmitted infection (STI) in the last six months (49.2%), with the highest proportion being reported among young women (69.4%). About one third of participants (34.0%) reported having used post-exposure prophylaxis (PEP) within the last six months, with female sex workers reporting the lowest proportion (18.9%) and the highest proportion among Young Women participants (48.2%). A small proportion of participants reported having shared injecting materials (5.9%), although the highest proportion was among LGBT participants (11.4%).

### 3.3. Vaccine Confidence Index

When answering the 5-point Likert scale VCI questions, participants mostly reported relatively high confidence in vaccines. The highest VCI scores were reported among young women (4.1, SD: 0.6), and female sex workers (4.1, SD: 0.7), followed by the LGBT subgroup (3.9, SD: 0.9). (See Table 3).

### 3.4. Split-Sample Mixed Logit Analysis

The regression analysis comparing conditional and mixed logit models, estimated both including and excluding the opt-out (i.e., the ‘None’ choice), reveals that mixed logit models, especially without opt-outs, captured greater preference heterogeneity, with higher pseudo R-squared and improved fit (lower AIC/BIC). Due to this finding, the mixed logit regression without opt-out questions was run for the split sample analysis. Notably, models with opt-outs showed heightened preferences for attributes such as longer protection (12 months) and private delivery, indicated by larger coefficients, especially in the mixed logit model.

The mixed logit regression model had a significant *p*-value (*p* < 0.001) for the standard deviation of the random parameter, the ASC, indicating evidence of unobserved heterogeneity in the data. The ASC findings suggested a left bias in the full sample (*β*: 0.08, 95% CI: 0.02, 0.14) and among female sex worker participants, indicating a significantly higher likelihood of choosing the option on the left (the first alternative) over the option on the right, when presented with a choice task (*β*: 0.14, 95% CI: 0.04, 0.23). No significant bias was observed for the young women or LGBT subgroups.

In the subgroup analysis, lower cost was strongly preferred across groups, with negative coefficients for all young women, female sex workers and LGBT groups (*β*: −0.29, −0.19, −0.21, respectively; all *p* ≤ 0.001). All groups also showed a strong preference for higher efficacy (per 30%) with positive coefficients for effectiveness (*β*: 0.41, 0.38, and 0.39, respectively; all *p* ≤ 0.001). The severity of side effects had a consistent negative impact, with severe side effects strongly reducing utility across groups, especially among female sex workers (*β*: −0.79) and LGBT individuals (*β*: −0.63). Duration of protection yielded mixed responses: the 12-month duration was significantly preferred by both young women (*β*: 0.33, *p* ≤ 0.001) and female sex workers (*β*: 0.34, *p* ≤ 0.001), while the LGBT group showed no significant preference. Confidentiality preferences varied: female sex workers and young women preferred private health facility settings for administration (*β*: 0.15, *p* ≤ 0.05; *β*: 0.10, *p* > 0.05), whereas the LGBT group displayed no strong preference for different levels of confidentiality settings (Table 4).

**Figure 3:**
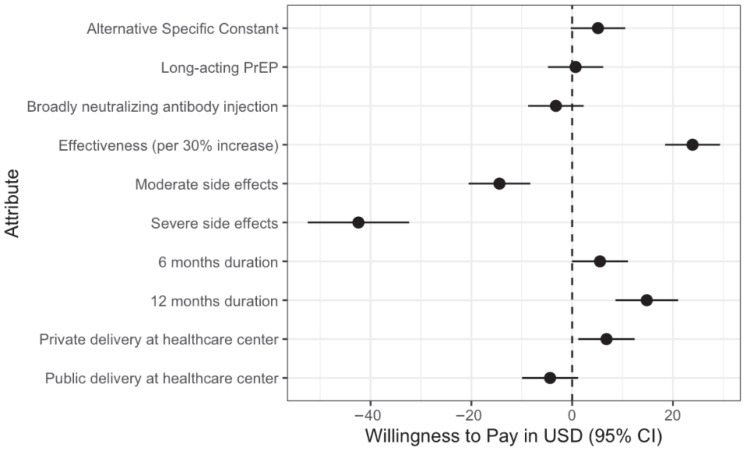
Willingness to pay for different attributes in the HIV DCE Study based on mixed logit regression findings.

All willingness-to-pay (WTP) values were derived using a baseline cost of USD 50 (equivalent to 183,500 UGX, assuming an exchange rate of USD 1 = 3670 UGX). Cost coefficients were modeled per 50,000 UGX (USD ~13.64) increment and converted to USD to facilitate interpretation. When looking at the full sample, participants’ willingness-to-pay (WTP) values indicated a strong preference for HIV prevention interventions with higher effectiveness (per 30% increase, WTP: $23.90, 95% CI: 18.43, 29.36), longer duration (12 months, WTP: $14.82, 95% CI: 8.59, 21.04), and private healthcare delivery (WTP: $6.82, 95% CI: 1.22, 12.42), while interventions with moderate (WTP: −$42.41, 95% CI: −52.48, −32.35) and severe (WTP: −$14.42, 95% CI: −20.56, −8.29) side effects showed reduced WTP (Figure 3).

In the analysis of prospective percentage uptake and marginal willingness to pay (WTP), the injectable bNAbs option had the highest uptake at 33.4% and a WTP of $66.39. Lenacapavir, the only option with 100% effectiveness but with severe side effects and a six-month duration, had a 23.8% uptake, with a higher WTP of $78.27. The HIV vaccine, which provides three years of protection with mild side effects and 75% effectiveness, showed a 25% uptake rate with the highest WTP at $81.38. In contrast, CAB-LA, effective for three months with 79% effectiveness and moderate side effects, showed the lowest uptake at 16.6% and a WTP of $56.01. There was a slightly reduced willingness to pay for public health center delivery compared to the private methods, though not significant (Table 5). We recognize that these estimates may be overestimated since they are based on the dataset without the opt outs, which may overemphasize the population’s willingness to pay and prospective uptake.

### 3.5. Sensitivity Analysis Findings

In the sensitivity analyses by help and income level, the likelihood ratio test did indicate significant differences based on the amount of help received by participants (*p* < 0.05), but not by income level (*p* = 0.12). Additional sensitivity analysis findings are reported in the Appendix A (Table A1, Table A2, Table A3 and Table A5).

## 4. Discussion

In this study, we found that effectiveness and side effects appeared to have the largest impact on the stated preference of a future preventative HIV injectable product, followed by a moderate effect of duration. It is important to note that these findings should be interpreted with caution, since it may over emphasize the importance of cost and effectiveness which were shown towards the top of the list, and effect sizes may be amplified due to the lack of inclusion of the opt out in the final paper. The degree of confidentiality only appeared to have an impact on preferences when comparing a private health facility with private at home. This was expected to be significant for LGBT populations, but it was not. The study found no significant difference in preference between different types of injection products, which may be explained by a lack of familiarity with the products; however, there was a slightly lower preference for the broadly neutralizing injection. The WTP and prospective uptake rates findings suggested that extended protection, higher effectiveness, and manageable side effects are key determinants in both uptake and economic valuation among participants.

We expected to see significant preferences among LGBT populations for a confidential site due to the ongoing Anti-homosexuality Act, increasing the stigma they must face in pursuing preventative options and putting them at added risk [30]. However, there were no significant preferences for a confidential location for this group. This may be because other attributes were more important to them and they still have a higher degree of trust in health care providers to maintain confidentiality as compared to other groups, or possibly because the levels or alternatives were not well defined. Other studies did find administration at a health facility rather than at a pharmacy was preferred [31]. The same study, conducted among youth in South Africa, found that longer duration of effectiveness and injectable administration were significantly associated with the choice to take a prevention product. Two stated preference studies among key populations in South Africa similarly found that longer lasting PrEP options were preferable to those that did not last as long [32,33]. Another study in Kenya and South Africa found a preference for higher efficacy as well as multipurpose technologies that both prevent HIV and pregnancy [32].

One of the strengths of this study is its opportune timing and its focus on three different subpopulations that are often underrepresented in research. Another strength of the study was that it involved LGBT participants and gathered their preferences and views during a sensitive period when many may have shied away from including them in their research. This may provide key information to help understand preferences for HIV preventative products among populations facing stigmatizing and criminalizing laws.

Limitations of the study include that the order of the presentation of attributes in the alternatives presented to participants was always the same, which may have introduced primacy bias, i.e., possibly causing the participants to value the attributes towards the top more than those at the bottom, due to the large number of attributes. Additionally, we were not able to gender match research assistants and participants as often as intended due to some logistical issues and therefore some of the more sensitive information may have been underreported (such as anal sex behaviors). This may have limited our ability to provide a more complete picture of older members of the LGBT and sex worker populations. Future studies should seek to engage older LGBT and sex worker populations to explore differences in preferences compared with younger populations. The sample size for the Young Women group was smaller than the rule of thumb calculation recommended, of at least 111, which reduced the power for that subpopulation’s analysis below what we had calculated a priori, due to the high overlap between the group with that of Female Sex Workers. The average age of the sample in this study was relatively young, reflecting not only the age limitation for the young women subgroup but also challenges with recruitment among intersecting populations.

Significant differences were found between the groups who received different amounts of help (Table A4), which might be explained by differences in participants’ characteristics, such as education level or other sociodemographic factors. Those who answered questions more independently may have also experienced less interviewer bias. For instance, participants may have answered sensitive questions more honestly, while those helped by the interviewer may have been affected by social desirability or acquiescence bias. However, participants who answered questions in the survey more independently may have also succumbed to sufficing (i.e., answering questions in the easiest way possible, rather than as honestly as possible), which may have also biased the data. We did not find significant differences by income, possibly because of a lack of statistical power. There was also evidence of unobserved heterogeneity in the model, as indicated in the findings from the mixed logit regression, which may have also accounted for some of those differences.

When looking at the results from the two-step opt out questions, this study demonstrated that there may be a high interest in a long-acting injection option, as a majority of participants chose not to opt out, but this finding may reflect social desirability bias in opting in. It may also be explained by the status quo bias, a bias where participants prefer what they already have in the current state (i.e., preferring the option they chose in the first step of the task). In other words, when asked to choose between getting the injection they previously selected or opting out, participants may have been biased to maintain their initial choice (the option they chose first), but in real life, they may choose otherwise.

Future studies should collect more specific information about gender identity to better understand the differences between subgroups within this larger group of different queer identities. Future research should also consider exploring analyses such as latent class analysis or hierarchical Bayesian modeling to better capture preference heterogeneity, especially when using larger sample sizes. While there are studies that have examined how the release of new long-acting injectable PrEP products may impact health in Sub-Saharan Africa, future studies may use DCE findings to project future uptake rates for different injectable profiles and integrate those into modeling studies, to explore impacts on HIV rates at different times of release, and therefore assess their added benefit [34,35]. Finally, additional implementation studies have the potential to bridge know-do gaps to ensure that effective injectable products are scaled up effectively in different contexts [36].

The findings from this study emphasize the importance of ensuring that products that reach these populations minimize side effects to ensure uptake. Additionally, policies may have a higher impact by prioritizing the distribution of subsidized and more effective HIV prevention injections to incentivize utilization of these technologies and protect the health and wellbeing of key and priority populations. They may also consider ensuring that messaging appropriately showcases minimal side effects and high efficacy rates to encourage uptake among key populations of interest in this study. Finally, this study highlights the importance of strategically rolling out and marketing different injections, particularly when multiple products enter the market simultaneously, as different profiles may be preferable and therefore have higher uptake for different key populations.

## 5. Conclusions

There is growing promise that multiple effective long-acting HIV prevention injections will soon be released for populations at increased risk for HIV in Uganda. According to these initial findings and other studies, it appears that these injectable prevention products may be well-received by the populations who need them [10,37]. Effectiveness, side effects and cost were identified as the main factors most likely to impact preferences regarding uptake in the future. There were also slight differences between different subpopulations, such as young women having a higher preference for free or low-cost HIV prevention injections. With the release of these new preventative injections, it is vital to consider how best to tailor techniques to promote them to the populations most in need. Future studies should also focus on understanding how intersecting identities may impact preferences. Future health promotion and messaging should emphasize effectiveness and be transparent about potential side effects when promoting injectables in the future. This study highlights the importance of strategically rolling out and marketing different injections if introduced simultaneously in the market, as different profiles may be preferable to different groups and therefore have higher uptake across key populations.

## Figures and Tables

**Table 1:** Attributes and levels used in the HIV preventative injectable discrete choice experiment.

	Level 1	Level 2	Level 3
Injection type	HIV Vaccine	Long-acting PrEP	Injectable bNAbs
Cost	Free	50,000 UGX	100,000 UGX
Effectiveness	30% Effective	60% Effective	90% Effective
Side Effects	Mild Side Effects	Moderate Side Effects	Severe Side Effects
Duration of Effectiveness	3 Months	6 Months	1 Year
Degree of Confidentiality	Private at Home	Private at a Healthcare Center	Public at a Healthcare Center

**Table 2:** Participant demographics in the HIV prevention injectable discrete choice experiment (*n* = 376).

	Young Women (*n* = 85)	Female Sex Workers (*n* = 159)	LGBT (*n* = 132)	Total (*n* = 376)	*p*-Value
**Site**					<0.001
Kampala	82 (96.5)	112 (70.4)	119 (90.2)	313 (83.2)	
Kayunga	3 (3.5)	27 (17.0)	0 (0)	30 (8.0)	
Mukono	0 (0)	20 (12.6)	13 (9.9)	33 (8.8)	
Age, mean (SD)	21.7 (1.9)	26.0 (7.6)	22.3 (3.1)	23.7 (5.7)	<0.001
**Sex**					<0.001
Male	0 (0)	0 (0)	77 (58.3)	77 (20.5)	
Female	85 (100.0)	159 (100.0)	30 (22.7)	274 (72.9)	
Other	0 (0)	0 (0)	23 (17.4)	23 (6.1)	
Refuse to respond	0 (0)	0 (0)	2 (1.5)	2 (0.5)	
**Marital status**					0.039
Single/Never married	86 (86.0)	123 (78.3)	104 (78.8)	292 (77.7)	
Married	6 (6.0)	11 (7.0)	22 (16.7)	54 (14.4)	
Divorced/ Separated/Widowed	6 (6.0	21 (13.4)	3 (2.3)	25 (6.7)	
Other	2 (2.0)	2 (1.3)	3 (2.3)	5 (1.3)	
**Tribe**					0.261
Muganda	49 (57.7)	93 (58.5)	96 (72.7)	238 (63.3)	
Munyankole	9 (10.6)	15 (9.4)	6 (4.6)	30 (8.0)	
Musoga	6 (7.1)	15 (9.4)	8 (6.1)	29 (7.7)	
Another Ugandan tribe	16 (18.8)	29 (18.2)	15 (11.4)	60 (16.0)	
Other nationality	5 (5.9)	7 (4.4)	7 (5.3)	19 (5.1)	
Religion					0.011
Catholic	29 (34.1)	41 (25.8)	27 (20.5)	97 (25.8)	
Other Christians	37 (43.5)	70 (44.0)	45 (34.1)	152 (40.4)	
Muslim	19 (22.4)	47 (29.6)	60 (45.5)	126 (33.5)	
Refused to respond	0 (0)	1 (0.6)	0 (0)	1 (0.3)	
**Able to read and write**	76 (89.4)	152 (95.6)	130 (98.5)	358 (95.2)	0.009
Education level *					0.018
No schooling	2 (2.4)	3 (1.9)	3 (2.3)	8 (2.1)	
Primary school	38 (44.7)	62 (39.0)	32 (24.2)	132 (35.1)	
Secondary/Vocational	40 (47.1)	84 (52.8)	78 (59.1)	202 (53.7)	
University	5 (5.9)	10 (6.3)	19 (14.4)	34 (9.0)	
**Employed**	65 (76.5)	123 (77.4)	96 (72.7)	284 (75.5)	0.641
**Number of people they provide for financially, ^ mean (SD)**	2.5 (0.7)	2.5 (0.9)	2.3 (1.0)	2.4 (0.9)	0.271
**Income per week (UGX), mean (SD)**	83.2 K (80.5 K)	79.2 K (100.2 K)	95.4 K (125.6 K)	85.8 K (106.1 K)	0.418
**Income per week in USD**	~22.75 (22.84)	21.66 (27.40)	26.09 (34.35)	23.46 (29.02)	

* Education: The level indicates that participants in that category completed at least some or all of that level of education.; ^ Excluding self. Note: We used an ANOVA to compare means for continuous variables, and the Pearson Chi-square test or Fisher’s exact test when cell frequencies were less than 5 to compare proportions between groups, with statistical significance defined as a *p*-value of less than 0.05.

**Table 3:** Participant Vaccine Confidence Index (VCI) responses in the HIV prevention injectable discrete choice experiment, Mean of 5-point Likert scale responses and Standard Deviation (*n* = 376).

	Young Women (*n* = 85), m (SD)	Female Sex Workers (*n* = 159)*,* m (SD)	LGBT (*n* = 132)*,* m (SD)	Total (*n* = 376)*,* m (SD)
Overall, I think vaccines are important for everyone to have. Overall, I think vaccines are safe.	4.5 (0.8)	4.5 (0.7)	4.4 (1.0)	4.5 (0.8)
	3.8 (1.3)	3.8 (1.2)	3.9 (1.2)	3.9 (1.2)
Overall, I think vaccines are effective.	4.0 (0.9)	3.9 (1.0)	3.8 (1.1)	3.9 (1.0)
Vaccines are compatible with my religious beliefs.	3.9 (1.2)	4.0 (1.1)	3.6 (1.4)	3.8 (1.3)
Vaccine Confidence Index Score * (out of 5):	4.1 (0.6)	4.1 (0.7)	3.9 (0.9)	4.0 (0.7)

Note: In the Likert Scale for each Vaccine Confidence Index Item, 1 = Strongly Disagree and 5 = Strongly Agree; * The VCI score represents the mean of all four items.

**Table 4:** Subgroup mixed logit regression analysis findings for participants regarding their preferences for HIV preventative injectables in Uganda (*n* = 376).

	Young Women (*n* = 85) *β* Coef, (95% CI)	Female Sex Workers (*n* = 159) *β* Coef, (95% CI)	LGBT (*n* = 132) *β* Coef, (95% CI)	All (*n* = 376) *β* Coef, (95% CI)
Alt. Sp. Constant	0.02 (−0.16, 0.20)	0.15 * (0.02, 0.28)	0.05 (−0.10, 0.20)	0.08 (−0.00, 0.17)
Type of injection (ref: HIV vaccine)				
Injectable PrEP	0.13 (−0.06, 0.32)	−0.04 (−0.18, 0.10)	−0.02 (−0.17, 0.13)	0.01 (−0.08, 0.10)
bNAbs injection	−0.10 (−0.29, 0.09)	−0.02 (−0.16, 0.12)	−0.07 (−0.22, 0.08)	−0.05 (−0.14, 0.04)
Cost (per 50,000 UGX/USD 13.64)	−0.29 *** (−0.38, −0.19)	−0.19 *** (−0.26, −0.12)	−0.21 *** (−0.28, −0.13)	−0.22 *** (−0.27, −0.17)
Effectiveness (per 30%)	0.41 *** (0.31, 0.51)	0.38 *** (0.31, 0.45)	0.39 *** (0.31, 0.47)	0.39 *** (0.34, 0.44)
Side effect severity (ref: mild side effects)				
Moderate	−0.19 * (−0.38, 0.00)	−0.27 *** (−0.41, −0.14)	−0.22 ** (−0.37, −0.07)	−0.24 *** (−0.33, −0.15)
Severe	−0.64 *** (−0.83, −0.45)	−0.79 *** (−0.93, −0.65)	−0.63 *** (−0.78, −0.48)	−0.69 *** (−0.78, −0.60)
Duration (ref: 3 months)				
6 months	0.10 (−0.09, 0.29)	0.24 *** (0.11, 0.38)	−0.05 (−0.20, 0.10)	0.09 * (0.00, 0.18)
12 months	0.33 *** (0.14, 0.52)	0.34 *** (0.20, 0.48)	0.08 (−0.07, 0.23)	0.24 *** (0.15, 0.33)
Confidentiality (ref: Private at home)				
Private at health facility	0.10 (−0.08, 0.28)	0.15 * (0.01, 0.29)	0.07 (−0.08, 0.22)	0.11 * (0.02, 0.20)
Public at health facility	−0.06 (−0.25, 0.13)	−0.11 (−0.25, 0.03)	−0.04 (−0.19, 0.12)	−0.07 (−0.16, 0.02)
AIC, BIC	1376.521, 1444.93	2559.997, 2635.921	2171.068, 2244.758	6087.81, 6174.063
Log likelihood	−676.26034	−1267.9984	−1073.5338	−3031.9052
Number of observations	2210	4134	3432	9776

* *p* ≤ 0.05; ** *p* ≤ 0.01; *** *p* ≤ 0.001; Likelihood ratio test: *p* > 0.05.

**Table 5:** Willingness to pay and prospective percentage uptake for different types of injectable bundles (*n* = 376).

Injection Option	Prospective Percentage Uptake *	Willingness to Pay (in USD) * (95% CI)
CAB-LA: 79% effective, lasts 3 months, moderate side effects, private administration at clinic	16.6%	56.01 (44.02, 68.00)
Lenacapavir: 100% effective, lasts 6 months, severe side effects, private administration at clinic	23.8%	78.27 (62.01, 94.53)
HIV Vaccine: 75% effective, lasts 3 years, mild side effects, private administration at clinic	25.0%	81.38 (63.02, 99.74)
Injectable bNAbs: 90% effective, lasts 1 year, moderate side effects, private administration at clinic	33.4%	66.39 (52.04, 80.80)

* In the prospective percentage uptake calculation, cost is held constant at USD 50 (~180,000 UGX).

## Data Availability

Due to the sensitivity of populations included in this study, the data is not publicly available, but a deidentified version of the data may be requested from the corresponding author, upon which we will review the request, from whom it is and how the requestor plans to use the data before sharing.

## Supplementary Materials

The following supporting information can be downloaded at: https://www.mdpi.com/article/10.3390/vaccines13111090/s1.

## Appendix A

**Table A1:** Sensitivity analysis using a split-sample conditional logit analysis to compare findings by weekly income regarding their preferences for HIV preventative injectables in Uganda (*n* = 376).

	≤50,000 UGX (*n* = 204) Coef, (95% CI)	>50,000 UGX (*n* = 172) Coef, (95% CI)	All (*n* = 376) Coef, (95% CI)
Alt. Sp. Constant	0.09 (−0.00, 0.18)	0.07 (−0.01, 0.15)	0.08 * (0.02, 0.14)
Type of injection (ref: HIV vaccine)			
Injectable PrEP	0.07 (−0.06, 0.20)	−0.03 (−0.15, 0.08)	0.01 (−0.07, 0.10)
bNAbs injection	0.03 (−0.10, 0.16)	−0.11 * (−0.22, 0.01)	−0.05 (−0.13, 0.04)
Cost (per 50,000 UGX/USD 13.64)	−0.17 *** (−0.23, −0.11)	−0.23 *** (−0.29, −0.17)	−0.20 *** (−0.25, −0.16)
Effectiveness (per 30%)	0.38 *** (0.32, 0.45)	0.35 *** (0.29, 0.41)	0.36 *** (0.32, 0.41)
Side effect severity (ref: mild side effects)			
Moderate	−0.26 *** (−0.39, −0.14)	−0.17 ** (−0.29, −0.06)	−0.21 *** (−0.30, −0.13)
Severe	−0.80 *** (−0.93, −0.67)	−0.52 *** (−0.64, −0.40)	−0.64 *** (−0.73, −0.56)
Duration (ref: 3 months)			
6 months	0.07 (−0.05, 0.20)	0.08 (−0.03, 0.20)	0.08 (−0.01, 0.16)
12 months	0.25 *** (0.12, 0.38)	0.21 *** (0.10, 0.32)	0.23 *** (0.14, 0.31)
Confidentiality (ref: Private at home)			
Private at health facility	0.10 (−0.02, 0.23)	0.08 (−0.04, 0.19)	0.09 * (0.01, 0.18)
Public at health facility	−0.08 (−0.21, 0.04)	−0.06 (−0.17, 0.06)	−0.07 (−0.15, 0.02)
Pseudo R^2^	0.0789	0.1129	0.0920
AIC, BIC	3408.285, 3480.623	2771.849, 2842.311	6174.637, 6253.701
Log likelihood	−1693.1426	−1374.9246	−3076.3184
Number of observations	5304	4472	9776

* *p* ≤ 0.05; ** *p* ≤ 0.01; *** *p* ≤ 0.001; Likelihood ratio test: *p* > 0.05.

**Table A2:** Sensitivity analysis using a split-sample conditional logit analysis to compare findings by amount of help received regarding their preferences for HIV preventative injectables in Uganda (*n* = 376).

	No Help (*n* = 109) Coef, (95% CI)	Some Help (*n* = 214) Coef, (95% CI)	a Lot of Help (*n* = 53) Coef, (95% CI)	All (*n* = 376) Coef, (95% CI)
Alt. Sp. Constant	0.08 (−0.03, 0.20)	0.07 (−0.01, 0.15)	0.09 (−0.07, 0.25)	0.08 * (0.02, 0.14)
Type of injection (ref: HIV vaccine)				
Injectable PrEP	0.02 (−0.14, 0.18)	−0.01 (−0.12, 0.10)	0.09 (−0.14, 0.31)	0.01 (−0.07, 0.10)
bNAbs injection	0.03 (−0.13, 0.19)	−0.14 * (−0.26, −0.03)	0.15 (−0.08, 0.38)	−0.05 (−0.13, 0.04)
Cost (per 50,000 UGX/USD 13.64)	−0.29 *** (−0.37, −0.20)	−0.20 *** (−0.26, −0.14)	−0.07 (−0.18, 0.04)	−0.20 *** (−0.25, −0.16)
Effectiveness (per 30%)	0.38 *** (0.30, 0.46)	0.36 *** (0.30, 0.42)	0.38 *** (0.27, 0.50)	0.36 *** (0.32, 0.41)
Side effect severity (ref: mild side effects)				
Moderate	−0.21 ** (−0.37, −0.05)	−0.17 ** (−0.28, −0.06)	−0.38 *** (−0.60, −0.15)	−0.21 *** (−0.30, −0.13)
Severe	−0.49 *** (−0.65, −0.33)	−0.73 *** (−0.84, −0.61)	−0.66 *** (−0.89, −0.43)	−0.64 *** (−0.73, −0.56)
Duration (ref: 3 months)				
6 months	−0.01 (−0.16, 0.15)	0.13 * (0.02, 0.24)	0.01 (−0.22, 0.23)	0.08 (−0.01, 0.16)
12 months	0.25 ** (0.09, 0.41)	0.20 ** (0.09, 0.31)	0.30 * (0.07, 0.52)	0.23 *** (0.14, 0.31)
Confidentiality (ref: Private at home)				
Private at health facility	0.02 (−0.14, 0.17)	0.08 (−0.03, 0.19)	0.30 ** (0.07, 0.53)	0.09 * (0.01, 0.18)
Public at health facility	−0.13 (−0.29, 0.03)	−0.13 * (−0.24, −0.02)	0.31 ** (0.08, 0.53)	−0.07 (−0.15, 0.02)
Pseudo R^2^	0.0952	0.1014	0.0979	0.0920
AIC, BIC	1799.424, 1864.868	3487.654, 3560.518	883.6569, 941.1692	6174.637, 6253.701
Log likelihood	−888.71206	−1732.8268	−430.82845	−3076.3184
Number of observations	2834	5564	1378	9776

* *p* ≤ 0.05; ** *p* ≤ 0.01; *** *p* ≤ 0.001; Likelihood ratio test: *p* > 0.05.

**Table A3:** Sensitivity analysis comparing conditional and mixed logit regression findings with and without opt out data regarding their preferences for HIV preventative injectables in Uganda (*n* = 376).

	Without Opt Outs		With Opt Outs	
	Conditional Logit (*n* = 376) Coef, (95% CI)	Mixed Logit (*n* = 376) Coef, (95% CI)	Conditional Logit (*n* = 376) Coef, (95% CI)	Mixed Logit (*n* = 376) Coef, (95% CI)
Alt. Sp. Constant	0.08 * (0.02, 0.14)	0.08 (−0.00, 0.17)	0.26 *** (0.21, 0.32)	0.28 *** (0.17, 0.39)
Standard deviation		0.60 *** (0.50, 0.70)		0.94 *** (0.85, 1.03)
Type of injection (ref: HIV vaccine)				
Injectable PrEP	0.01 (−0.07, 0.10)	0.01 (−0.08, 0.10)	0.16 *** (0.09, 0.24)	0.19 *** (0.11, 0.27)
bNAbs injection	−0.05 (−0.13, 0.04)	−0.05 (−0.14, 0.04)	0.11 ** (0.03, 0.18)	0.13 ** (0.04, 0.21)
Cost (per 50,000 UGX/USD 13.64)	−0.20 *** (−0.25, −0.16)	−0.22 *** (−0.27, −0.17)	−0.01 (−0.04, 0.03)	−0.02 (−0.05, 0.02)
Effectiveness (per 30%)	0.36 *** (0.32, 0.41)	0.39 *** (0.34, 0.44)	0.43 *** (0.40, 0.46)	0.51 *** (0.47, 0.55)
Side effect severity (ref: mild side effects)				
Moderate	−0.21 *** (−0.30, −0.13)	−0.24 *** (−0.33, −0.15)	−0.05 (−0.12, 0.03)	−0.03 (−0.11, 0.05)
Severe	−0.64 *** (−0.73, −0.56)	−0.69 *** (−0.78, −0.60)	−0.40 *** (−0.47, −0.32)	−0.45 *** (−0.53, −0.37)
Duration (ref: 3 months)				
6 months	0.08 (−0.01, 0.16)	0.09 * (0.00, 0.18)	0.20 *** (0.13, 0.27)	0.24 *** (0.16, 0.33)
12 months	0.23 *** (0.14, 0.31)	0.24 *** (0.15, 0.33)	0.34 *** (0.27, 0.42)	0.40 *** (0.31, 0.48)
Confidentiality (ref: Private at home)				
Private at health facility	0.09 * (0.01, 0.18)	0.11 * (0.02, 0.20)	0.22 *** (0.14, 0.29)	0.27 *** (0.19, 0.35)
Public at health facility	−0.07 (−0.15, 0.02)	−0.07 (−0.16, 0.02)	0.08 * (0.00, 0.15)	0.10 * (0.02, 0.18)
Pseudo R^2^	0.0920		0.1820	
AIC, BIC	6174.637, 6253.701	6087.81, 6174.063	11,107.86, 11,194.55	10,328.75, 10,423.32
Log likelihood	−3076.3184	−3031.9052	−5542.9308	−5152.3732
Number of observations	9776	9776	19,552	19,552

* *p* ≤ 0.05; ** *p* ≤ 0.01; *** *p* ≤ 0.001.

**Table A4:** Distribution of amount of help received completing the survey by subpopulation (*n* = 376).

	No Help (*n* = 109)	Some Help (*n* = 214)	A Lot of Help (*n* = 53)
Young Women (*n* = 85)	25 (29.4%)	52 (61.2%)	8 (9.4%)
Female Sex Workers (*n* = 159)	37 (23.3%)	94 (59.1%)	28 (17.6%)
LGBT (*n* = 132)	47 (35.6%)	68 (51.5%)	17 (12.9%)

**Table A5:** Willingness to pay and prospective percentage uptake for different types of injectable bundles using mixed logit regression findings with opt out (*n* = 376).

Injection Option	Prospective Percentage Uptake *	Willingness to Pay (in USD) *
CAB-LA: 79% effective, lasts 3 months, moderate side effects, private administration at clinic	16.6%	56.01
Lenacapavir: 100% effective, lasts 6 months, severe side effects, private administration at clinic	23.8%	78.27
HIV Vaccine: 75% effective, lasts 3 years, mild side effects, private administration at clinic	25.0%	81.38
Injectable bNAbs: 90% effective, lasts 1 year, moderate side effects, private administration at clinic	33.4%	66.39

* In the prospective percentage uptake calculation, cost is held constant at USD 50 (~180,000 UGX).
