# Supplementary material for: Finding the Sweet Spot: Preferences for Effectiveness, Duration, and Side Effects in a Discrete Choice Experiment Among Uganda’s Key Populations
Source: Vaccines (Basel). 2025 Oct 24;13(11):1090. doi: 10.3390/vaccines13111090 (PMC12656451; doi:10.3390/vaccines13111090)
Supplement: Supplementary file 1 [file vaccines-13-01090-s001.zip › DIRECT_Checklist_HIV DCE_20251013.pdf]

DIRECT Reporting Checklist for Discrete Choice Experiments

Manuscript Title: Finding the Sweet Spot: Preferences for Effectiveness, Duration, and Side Effects in a Discrete Choice Experiment Among Uganda's Key Populations

Corresponding Author: Maiya G. Block Ngaybe, Mel and Enid Zuckerman College of Public Health, University of Arizona

Checklist based on: Ride et al., 2024, PharmacoEconomics

Section	Item	Description and How It Is Reported in This Study
1. Title and Abstract	1.1	Title identifies the study as a discrete choice experiment (DCE). Reported: "Preferences for Effectiveness, Duration, and Side Effects in a Discrete Choice Experiment…"
	1.2	Abstract includes objectives, methods, key attributes, sample, analysis, and main findings. Present.
2. Introduction	2.1	States rationale for using DCE and research question. "This study used a DCE to determine preferences for preventative HIV injectables…"
	2.2	Describes relevance to health policy and decision-making. "Findings inform policymakers on cost, side effects, and effectiveness priorities."
3. Methods – Design	3.1	Theoretical foundation described (Lancaster's value theory and random utility theory). Reported.
	3.2	Attributes and levels identification process described (literature, expert consultation, qualitative interviews). Reported, Table 1.

	3.3	Choice task design and experimental structure explained, including blocking and number of tasks. “13 tasks per block; balanced overlap design.”
	3.4	Opt-out or “none” option inclusion and rationale stated. “Two-step question included opt-out after choice A/B.”
	3.5	Description of software used. “Lighthouse Studio v9.15.6.”
4. Methods – Participants and Data Collection	4.1	Target population defined. “Female sex workers, LGBT individuals, young women 18–24.”
	4.2	Recruitment and sampling method detailed (purposive and respondent-driven). Section “Study Population.”
	4.3	Sample size justification provided (Orme’s rule of thumb). Present.
	4.4	Ethical approval and informed consent procedures described. Section “Ethics Statement.”
5. Methods – Experimental Procedure	5.1	Description of survey preamble and comprehension checks. Reported (Luganda translation, pilot test).
	5.2	Presentation format of choice tasks (images, two-alternative format) specified. “Visual graphics by local artist.”
6. Methods – Analysis	6.1	Utility specification given, including variables and reference categories. Table 4 and text.
	6.2	Model type and estimation

		method stated (mixed logit with random ASC). Reported.
	6.3	Accounting for repeated choices and heterogeneity explained. Random ASC + panel data structure.
	6.4	Handling of opt-out responses described and compared. Table A3 (models with and without opt-outs).
	6.5	Sensitivity and subgroup analyses described. Tables A1–A2 (income and help-level subgroups).
	6.6	WTP estimation method explained. "Delta Method" noted in Data Analysis section.
	6.7	Software for analysis stated (Stata v18, Lighthouse Studio). Present.
7. Results	7.1	Descriptive statistics and participant characteristics provided. Table 2.
	7.2	Main model results (coefficients, SEs, CIs) presented. Table 4.
	7.3	WTP results reported with 95% CIs and cost conversion explained. Table 5 and clarified sentence added.
	7.4	Scenario-based uptake predictions or policy simulations described. Table 5 prospective uptake.
	7.5	Sensitivity and subgroup results reported. Appendices A1–A3.
8. Discussion	8.1	Interpretation of main findings in context. Section "Discussion."
	8.2	Discussion of limitations (e.g., primacy bias, small

		subgroup). Present.
	8.3	Implications for policy and future research stated. "Policy recommendations on pricing and messaging."
9. Ethics and Transparency	9.1	Ethics approval and consent reported. SPH-2023-465/HS 3769ES.
	9.2	Funding, data availability, and conflicts disclosed. "Fogarty/NIH GHES Fellowship" and full statements.
10. Overall Reporting Quality	10.1	Checklist explicitly mentioned in Methods. "The DIRECT checklist was used to guide reporting."
